# A comparative evaluation of shear bond strength between feldspathic porcelain and lithium di silicate ceramic layered to a zirconia core– An *in vitro* study

**DOI:** 10.4317/jced.57569

**Published:** 2020-11-01

**Authors:** Aghin Moses, Lambodaran Ganesan, Sathya Shankar, Annapoorni Hariharan

**Affiliations:** 1 Post graduate student. Department of prosthodontics, Faculty of dentistry, Meenakshi Academy of Higher Education and Research, Chennai; 2 MDS, Associate Professor. Department of prosthodontics, Faculty of dentistry, Meenakshi Academy of Higher Education and Research, Chennai; 3 Assistant Professor. Department of prosthodontics, Faculty of dentistry, Meenakshi Academy of Higher Education and Research, Chennai; 4 MDS, Prof & Head. Department of prosthodontics, Faculty of dentistry, Meenakshi Academy of Higher Education and Research, Chennai

## Abstract

**Background:**

The bond strength between the zirconia core and ceramic veneer is the weakest component in the layered structure. Delamination of veneering ceramic is reported as one of the most frequent problems associated with Veneered Zirconia restorations. The aim of this study is to compare the shear bond strength of lithium di silicate porcelain to that of feldspathic porcelain on a zirconia Substrate.

**Material and Methods:**

Two groups (group A and B) of zirconia blocks with each group having 20 samples were fabricated according to Schmitz Schulmeyer method. Group A (n =20 ) samples were veneered with feldspathic veneering porcelain and Group B (n=20) samples were veneered with heat pressed lithium disilicate ceramic. The fabricated samples were then evaluated for shear bond strength in Universal Testing Machine. The values were then statistically analyzed using independent sample t-test.

**Results:**

Results of the current study showed that mean shear bond strength of feldspathic porcelain 11.40±1.29 MPa is comparatively lower than the mean shear bond strength of the lithium disilicate group 18.81±1.76 MPa. The statistical analysis indicated that (*p* value<0.01) there is a statistically significant difference in the shear bond strength between the two groups.

**Conclusions:**

The heat pressed lithium disilicate veneering materials has a better shear bond strength compared to feldspathic veneering ceramic material when layered to a zirconia core and it can be used as a viable alternative material to feldspathic porcelain layering material in bilayered zirconia restorations.

** Key words:**Zirconia, bilayered ceramics, lithium disilicate , shear bond strength, ceramic chipping.

## Introduction

The ultimate objective of any prosthodontic procedure is to restore function and aesthetics. In modern day dental practice, since the expectation of the patients are highly demanding, restoration of aesthetics plays a vital role in the outcome of the treatment. The evolution of dental ceramics in the past couple of decades have played a crucial role in achieving the same.

The term ceramic was derived from a Greek word “keramos” which means “burnt stuff”. Ceramics are non-metallic, inorganic, man made solid objects which are formed by baking raw materials at high temperatures. In dentistry the ceramics have been used in various situations for rehabilitation of missing teeth structures (1). They are used as inlays, onlays, overlays, laminate veneers, crowns, implant prosthesis etc. It has been proven to be a better choice than the resin restorations in terms of survival rates (2).

The ceramics were first used with base metal substructure to aid in strength and they are called as the metal ceramic restorations or porcelain fused metal restorations. The first successful porcelain fused metal restoration was documented in 1960s and has proven to be a gold standard in crown and bridge prosthodontics (3-5). Because of its aesthetic values and excellent strength properties, there was a rapid increase in the use of metal ceramics. Though the metal ceramic restorations had various advantages, they also had few disadvantages like decreased transmission of light through the restoration, discoloration of gingiva around the abutment teeth (6), allergic reactions and release of metallic ions in to the gingival tissue (7). To overcome these disadvantages, the all ceramic restorations or metal free ceramic restorations were introduced which does not have a base metal substrate or a noble metal substrate (1). The introduction of zirconia to dentistry have paved the way for fabricating prosthesis with superior properties and attaining excellent results (8).

Zirconia usually refers to zirconium oxide. According to the periodic Table, it is grouped under metal category but in dentistry it is considered as a kind of ceramic as its aesthetic properties are similar to ceramics. Garvie *et al.* called zirconium as “ceramic steel” because of its similar mechanical properties like metals (9). Zirconium oxide has three phases of existence. Pure zirconia exists as monoclinic phase (up to the temperature range of 1170ºC) and the other two phases are tetragonal phase (1170ºC to 2370ºC) and a cubic phase (from 2370ºC to 2680ºC). Once the temperature comes down, the phases are reversed automatically. Rare earth metal oxides such a magnesium oxide (MgO), calcium oxide (CaO) and yttrium oxide (Y2O3) are used to stabilize the zirconium from reverting back to the monoclinic phase and stabilized in a metastable state of tetragonal zirconia polycrystal and the Commonly used stabilizer in dental zirconia blocks is the yttrium oxide (10,11).

Besides having excellent mechanical properties, zirconia has a high refractory index which makes it critical to match the natural esthetics of the adjacent tooth difficult and hence layering of zirconia core is essential to give a more natural appearance (10). The commonest layering method followed is the powder slurry method with feldspathic porcelain. Though this bilayered zirconia restoration has been extensively used , the Chipping and delamination of veneering ceramic from zirconia core is one of the common modes of failure in this metal free ceramic restorations. where, the veneering ceramic debonds from the underlying core material (12,13). Studies in the literature have also shown that delaminations from the zirconia core ceramic (14,15)and minor chip-off fractures (16) of the veneering ceramic were one of the most frequent reason for failures of bilayered zirconia restorations.

Thus one of the primary factor influencing the long-term success of bilayered zirconia restorations is the weak performance of the veneering ceramics and its limited bond to the zirconia substrate. Clinical studies on the survival rates of the bilayered ceramic restorations in the literature showed that the Chip-off fracture rates at 15% after 24 months 16 , 25% after 31 months respectively (17). A review of the literature for FPD on ceramic chipping showed that it was about 54% for zirconia FDPs and 34% for PFM FDPs (17).

To overcome the problems with the veneered all ceramic crowns, monolithic crowns were tried, where one single block of zirconia is milled in to an anatomic crown form. By this means, veneer chipping can be avoided but the aesthetic problems such as shade matching and translucency can occur because of the crystalline nature (13). Another major drawback of the Monolithic zirconia crowns was its tendency to wear off the opposing teeth much faster when compared to veneering ceramics (18).

On the contrary the other variant in monolithic restoration , lithium disilicate ceramics finds its application in monolithic restorations without veneering ceramic because of its high translucency (19-21). However the monolithic lithium disilicate material did not perform well in case of posterior zone because of its compromised mechanical properties (19,20). Hence, a combination of zirconia as a core and lithium disilicate as a veneering material may provide a restoration with optimal esthetic and mechanical properties.

With this background, the present study was conducted to evaluate the shear bond strength between conventional feldspathic porcelain and lithium disilicate material, layered to a zirconia core.

## Material and Methods

**Figure 1:**
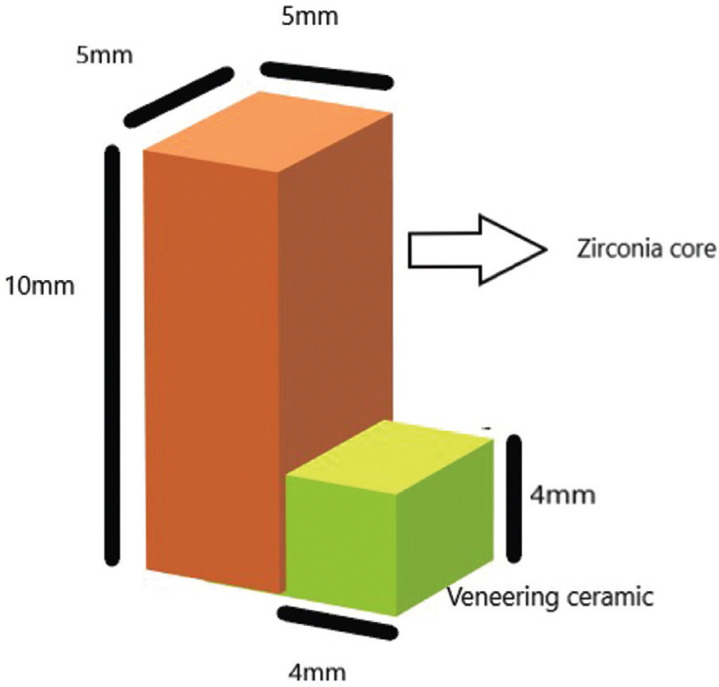
Schmitz Schulmyer model.

In this study all ceramic models were fabricated on the basis of “Schmitz–Schulmeyer test” (Fig. 1) and divided in to two groups namely “Group A” and “Group B”. Both the groups have a similar core material that is, the yttrium stabilized zirconia of standard size and it is layered with 2 different layering ceramics. In Group A, the layering ceramic is the feldspathic ceramic (Ivoclar IPS classic) coated by powder slurry method. In Group B, the layering ceramic is the hot-pressed lithium disilicate ceramic (IvIoclar IPS emax press).

-Fabrication of Zirconia block

A metal steel die was fabricated with a standard size well of 5mm deep, 10mm in length and 5 mm in width . Scanning of this well in the metal die was done in a digital scanner and a CAD image was obtained . Once the CAD image was obtained a small hole is placed on the die which passes through and through the well, for the easy removal of zirconia blocks after size verification. The scanned image is now milled in a CAD-CAM milling machine using a pre-sintered zirconia block. The milled zirconia block (Dentcare zirconia) of standard size (10mm Length x 5mm height x 5 mm width) is obtained. This milled block is verified in the metal die and can be retrieved by using a lecron carver, pressing the block through the circular hole that is placed on the die.

A sum of 40 zirconia samples were fabricated using the same method and all the blocks were verified using the metal die. Once all the twenty zirconia blocks were milled, they were categorized into 2 groups (Group A and Group B). Each group had 20 samples and all the zirconia blocks are cleaned up using ultrasonic cleaner for 10 minutes.

Group A: Feldspathic ceramic Layering.

**Figure 2:**
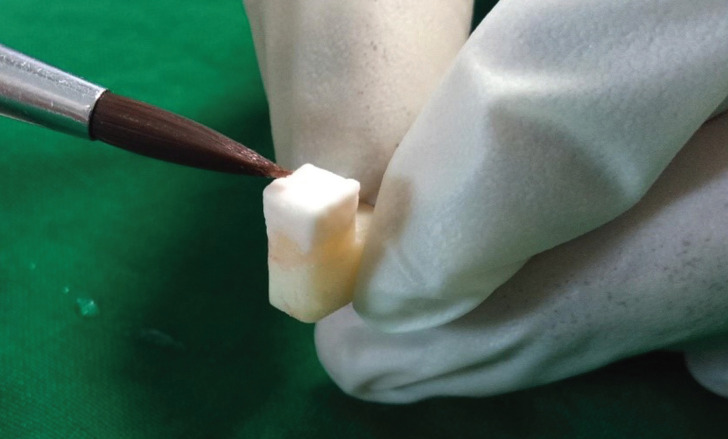
Layering of Feldspathic porcelain to zirconia core.

Layering of the zirconia blocks in Group A was done using conventional powder slurry method (Fig. 2). Zirconia is coated with a 0.1mm thick layer of zirliner (IPS e.Max Ceram zirliner) in the area pre-determined for layering ceramic and fired in a furnace at 960ºC according to manufacturer’s instructions. Feldspathic porcelain (IPS Classic) which is available as powder is mixed with water in a ratio according to the manufacturer’s instructions and coated in layers and sintered in a ceramic furnace at 920ºC. Two firing cycles was required to achieve the final size of the sample. The final size of the layering was 4mm (length), 5mm (width), 4mm (Height) on the zirconia block. The final size of the layering specimen was measured using Vernier calliper.

Group B: Lithium di silicate Layering

**Figure 3:**
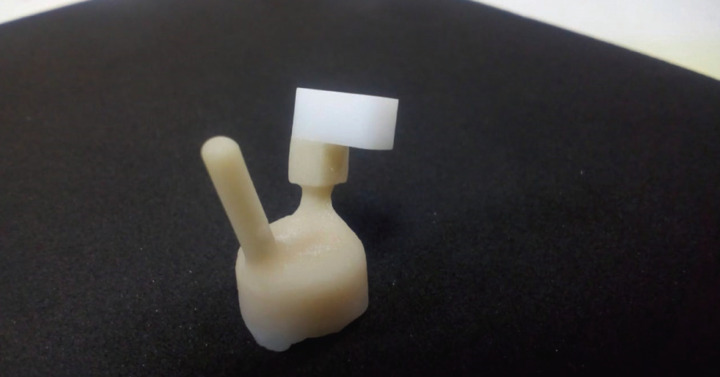
Heat pressed lithium di silicate layered sample.

Layering of the zirconia blocks in Group B was done using hot pressed lithium disilicate zirconia. The zirconia block was layered with 0.1mm thick layered zirliner (IPS e.Max Ceram zirliner) in the area pre-determined for layering ceramic and fired in a furnace at 960ºC according to manufacturer’s instructions. Once the firing is done the block is allowed to cool down and the wax pattern for the ceramic block is built using inlay wax to the same size, 4mm (length), 5mm (width), 4mm (Height) as in Group A. After the fabrication of wax pattern on the zirconia block, it is invested in to a phosphate bonded investment material (IPS Pressvest premium) for all ceramic restoration. Once the investment is set, it is kept for dewaxing in the furnace. After dewaxing, preheating of the investment material was done to a temperature of 403ºC and then the sintering unit (Programat EP 3000, Ivoclar)was heated up to 730ºC before loading of the investment and the hot press ceramic ingot (IPS e.max Press). The ingot was pressed automatically in to the mould cavity by the unit once 900ºC is reached, through the sprue channel (Fig. 3).Then the sprue channel was cut and the dimensions of the veneering materials were checked.

-Thermocycling:

The specimens of group A and group B underwent thermocycling . Thermocycling was done at a temperature between 5ºC - 55ºC.The specimens were initially kept in a water bath at 55ºC and then in a refrigerator at 7ºC, with an immersion time of 45 seconds in each and the cycle was repeated up to 20,000 times before the final testing of the specimens were done.

**Figure 4:**
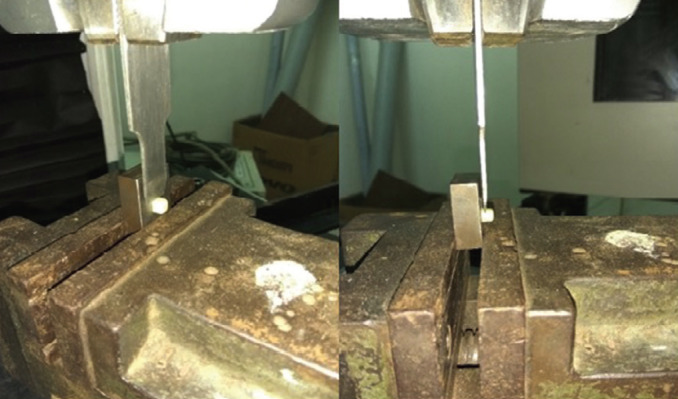
Shear bond strength testing using Universal testing machine.

Testing of shear bond strength:

Shear bond strength of the samples were assessed using Instron universal testing machine by applying static load. A chisel shaped sliding plate was used to load at the junction between the zirconia and the veneering ceramic with the crosshead speed of 2mm per minute (Fig. 4). The delamination of the layering ceramic occurred as a result of application of load. Both the load and the shear bond strength were recorded at the time when the delamination occured and the results were statistically analysed using Shapiro wilk test and independent sample T test.

## Results

The results of the study showed that the mean shear bond strength of group B i.e (18.80 mpa) the heat pressed lithium dilisilacte ceramics were higher when compared to group A i.e (11.40 mpa) feldspathic porcelain. Similarly the mean load causing the delamination of group B (376.16 N) was greater than that of group A samples (225.36 N) Table 1.

**Table 1:**
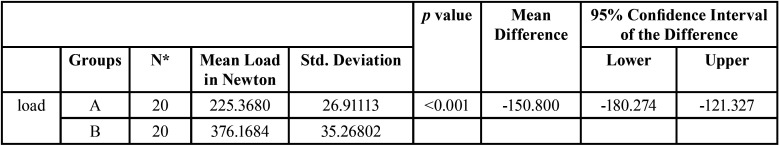
Comparison of load between two groups.

-Statistical Analysis

The data was checked for normality using Shapiro wilk test and the data was found to be normally distributed. Hence parametric tests of significance were used for the comparison of data. Intergroup comparison of shear bond strength was made using independent sample t-test ( Table 2). In the current study *P* value of <0.05 was considered statistically significant. The results of the statistical analysis indicated that the *p* value < 0.01, which shows that there is a statistically significant difference in the shear bond strength between the two groups. Mean difference in value of shear bond strength is found to be 7.40 mpa with highest mean value in group B.

**Table 2:**
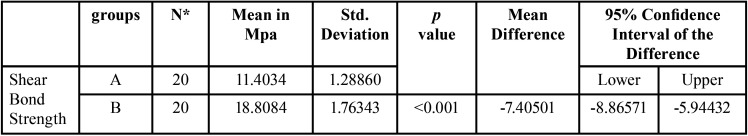
Comparison of shear bond strength between two groups.

## Discussion

Dental ceramics being one of the most common material of choice for enhancing esthetic needs in dentistry, has evolved so much in the recent times. In the current trend of metal free ceramics, bilayered zirconia crowns are more widely used than the monolithic zirconia crowns because of esthetic reasons. However the bilayered zirconia crown has a major disadvantage of ceramic chipping caused due to the difference in coefficient of thermal expansion, lesser shear bond strength and lesser tensile strength (22). It has been documented that the hot pressed ceramics is said to have very less or nil internal defects and therefore the incidence of delamination from the core material is less when compared to conventional method (23).On literature search till date, studies comparing the shear bond strength of manually layered feldspathic porcelain and hot-pressed lithium di silicate ceramics to zirconia core are minimal and hence this study.

In order to assess the shear bond strength, Schmitz–Schulmeyer test was adopted in the current study. Schmitz–Schulmeyer test is the planar interface shear bond test, used to assess the shear bond strength of the materials (23). Hammad *et al.* stated that, this test is the best method for measuring the bond strength as it requires minimal experimental variables and also this testing method results in a uniform interfacial stress by directly applying the force to the junction (24). Guess *et al.* also stated that Schmitz-schulmeyer *et al.* method can be used as an effective method for finding the shear bond strength (15).

In Schmitz-Schulmeyer test, standardized size of samples were made to avoid errors that can occur due to the change in the surface area that is bonded. Size of the zirconia blocks were standardized with 10 mm height, 5mm length and 5mm width. The size of veneering was standardized with 4mm height, 4mm length and 5mm width (23).Total surface area of bonding in the current study is standardized as 20mm2. Digital impression and CAD CAM fabricated restorations were used in this study, since the above mentioned methods have proven to produce good results compared to conventional impression methods(25). Impression of the metal die was made using a laboratory scanner and the resultant image was milled using in a CAD CAM milling machine.

Surface treatment of zirconia surface before layering is proven to have an impact on the bond strength of veneering ceramic. Matsumoto *et al.* (26), Yoon *et al.* (14) in their studies have proved that application of ceramic liner materials were beneficial in increasing the bond strength (14). Therefore, milled specimens were coated with zirliner which is a fusion ceramic acts as an intermediate layer between zirconia and the veneering ceramic. Application of liner will improve the adhesion between zirconium core and veneering ceramic by compensating for the discrepancy in the coefficient of thermal expansion and also by increasing the wettability of the zirconia surface (27).

Thermocycling was done under controlled temperatures to mimic the temperature changes happening in the oral environment. It has been stated that the temperature changes in a wet environment has a negative impact on the bond strength of the ceramics (14). Oral cavity being a dynamic environment with frequent temperature changes occurring due to various food items, it is wise to mimic the oral environment with thermocycling process to assess the long-term efficiency of the material in the oral cavity during function.

Kim *et al.*, Sim *et al.*, Fischer *et al.*, Guess *et al.* and various other authors have used static loading method in a universal testing machine for determining the shear bond strength assessment (15,23,27,28). Standardizing of all these methods are of paramount importance to avoid variations in the results.

The results of the current study showed that mean shear bond strength of feldspathic porcelain was 11.40±1.29 MPa which is comparatively lower than the mean shear bond strength of the lithium disilicate group 18.81±1.76 MPa. The *P* value was less than < 0.01 which represents that the difference in the shear bond strength between both the groups were statistically significant.

The results of the study were in accordance with the studies of Sim *et al.* (23), Subash *et al.* (29), Aboushelib *et al.* (30) who also concluded that hot pressed lithium disilicate ceramic had better shear bond strength when compared to conventional layering ceramics. The reason for this variation in the shear bond strength is due to the fact that in feldspatchic porcelain layering, the difference in the Coefficient of thermal expansion has a significant role in the shear bond strength (27,31). Since layering ceramics are heated at high temperatures at various stages and this process creates thermal residual stresses within the restoration after cooling down (11) and also causing phase transformation of zirconia resulting in surface uplifts which affects the bond strength (32-34). Further, the mismatch of young’s elastic modulus on application of masticatory forces adds on to the internal stresses and eventually end up in failure of the restoration (35,36).

The better bond strength of lithium di silicate is due to the fact that the ingot is pressed at high temperatures and the pressure is maintained till the cooling is complete which enhances the surface contact between the lithium di silicate veneer and the zirconia core. This enhanced contact during the cooling down of the material below the glass transition temperature (15,20) would have resulted in better shear bond strength between the veneering material and core zirconia. Also in heat pressed layering, the material undergoes minimal shrinkage when compared to feldspathic porcelain and less internal stresses (21) would have enhanced the bonding between the veneering material and the core ceramic.

The limitations of the study are that the test was not performed in anatomical crown and also it is *in vitro* study. The bond strength was tested between 2 planar surfaces of geometrical shapes. Since this is an *in vitro* study, all the dynamic changes happening in the oral environment cannot be replicated which could probably have an impact on the results that are obtained.

## Conclusions

Within the limitations of the current study, it can be concluded that

1.The heat pressed lithium disilicate veneering materials has a better shear bond strength when compared to feldspathic ceramic veneering material and hence the chances for ceramic chipping is less in lithium disilicate veneered zirconia restorations.

2. Due to it´s better shear bond, lithium disilicate veneered zirconia restorations can be used as a viable alternative material to feldspathic porcelain layering material and could prove to be the solution for chipping problems in bilayered zirconia restorations.
